# High‐Pressure Synthesis of Crystalline Double‐Layer Carbon Nitride Networks Stabilized in Bi_7_C_10_N_18_(N_3(1‐_ *_x_*_)_O_3_ *_x_*)

**DOI:** 10.1002/anie.202506406

**Published:** 2025-07-23

**Authors:** Lukas Brüning, Nityasagar Jena, Pascal L. Jurzick, Elena Bykova, Nico Giordano, Mohamed Mezouar, Igor A. Abrikosov, Maxim Bykov

**Affiliations:** ^1^ Institute for Inorganic and Analytical Chemistry Goethe University Frankfurt 60438 Frankfurt am Main Germany; ^2^ Department of Physics, Chemistry and Biology (IFM) Linköping University Linköping SE‐58183 Sweden; ^3^ Institute for Inorganic Chemistry University of Cologne 50939 Cologne Germany; ^4^ Institute of Geoscience Goethe University Frankfurt 60438 Frankfurt am Main Germany; ^5^ Deutsches Elektronen‐Synchrotron (DESY) 22607 Hamburg Germany; ^6^ European Synchrotron Radiation Facility (ESRF) Grenoble 38043 France

**Keywords:** 2D Materials, Carbonitrides, Diamond anvil cell, High‐pressure, Single‐crystal X‐ray diffraction

## Abstract

Application of high‐pressure conditions in chemical synthesis has proven to access a wide range of novel nitrogen‐rich compounds and to overcome the stability of the dinitrogen molecule. In the present work, we report the high‐pressure high‐temperature (HPHT) synthesis of Bi_7_C_10_N_18_(N_3(1‐_ *_x_*_)_O_3_ *_x_*), which features double‐layers of poly‐*N*‐(1,3,5‐triazin‐2‐*yl*)‐guanidine [C_4_N_6_ *^x^* ^‐^]*_n_* and non‐polymerized guanidinate anions CN_3_^5‐^. The structure model was determined by means of synchrotron single‐crystal X‐ray diffraction and is fully corroborated by theoretical calculations. The poly‐*N*‐(1,3,5‐triazin‐2‐*yl*)‐guanidine illustrates an example of a 2D polymerized anionic C─N network and represents the first intermediate between highly charged CN_3_^5−^ anions and fully condensed graphitic carbon nitride networks achieved by HPHT conditions. This opens a pathway to a widely varied family of hydrogen‐free nitridocarbonates, which has the potential to develop into an alternative synthesis route to classical polycondensation reactions.

## Graphical abstract

High‐pressure chemical reaction between Bi and various hydrogen‐free C─N precursors induced by the infrared laser heating in a diamond anvil cell leads to the formation of a compound Bi_7_C_10_N_18_(N_3(1‐_ *_x_*_)_O_3_ *_x_*), featuring double layers of poly‐*N*‐(1,3,5‐triazin‐2‐*yl*)‐guanidine [C_4_N_6_ *^x^* ^‐^]*_n_* and non‐polymerized guanidinate anions CN_3_^5‐^. The unprecedented 2D‐polymerized anionic C─N network represents the intermediate between highly charged CN_3_^5‐^ guanidinate anions and fully condensed graphitic carbon nitride networks.

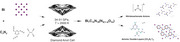

Exploring new methods to synthesize solid‐state nitrides is crucial because these materials may have a wide array of potential applications.^[^ ^1^, ^2^ ^]^ However, producing nitrogen‐rich compounds is challenging due to the strong N≡N bond of the dinitrogen molecule, resulting in thermodynamic instability of such compounds. Among the various synthesis techniques, high‐pressure methods offer a distinct advantage in creating compounds with exceptionally high nitrogen content, as demonstrated by preceding research on high‐pressure nitrides, pernitrides, and polynitrides.^[^ ^3^, ^4^, ^5^, ^6^, ^7^, ^8^, ^9^, ^10^, ^11^, ^12^, ^13^, ^14^ ^]^ The majority of these compounds were produced in laser‐heated diamond anvil cells (LH‐DACs) in direct reactions between the elements. Recent studies yielded novel binary nitrides of main group elements like C_3_N_4,_^[^ ^15^ ^]^ AsN,^[^ ^16^ ^]^ Sb_3_N_5_,^[^ ^17^ ^]^ and BiN^[^ ^18^ ^]^ and SN_2_.^[^ ^19^ ^]^ However, many of the binary nitrides, synthesized under high pressure, are not recoverable under ambient conditions, making a comprehensive analysis and application challenging. Stabilization of nitrogen‐rich compounds can be achieved by introducing strong bonding between nitrogen and other elements and by avoiding unfavorable single and double N─N bonds.^[^ ^20^, ^21^, ^22^ ^]^ Compared to nitridosilicates and nitridophosphates, nitridocarbonates can be much more structurally diverse due to the ability of carbon and nitrogen to form bonds of orders different from 1. The M─C─N system tends to form complex CN*_x_* anions with electron‐donating elements. Inorganic salts containing a metal, carbon, and nitrogen at ambient conditions are mostly represented by cyanides [C≡N]^−^ and carbodiimides [N═C═N]^2‒^, which feature carbon in *sp*‐hybridization. Changing the carbon hybridization from *sp* to *sp* ^2^ and *sp* ^3^ results in the guanidinate anion CN_3_^5−^ and the ortho‐nitridocarbonate anion CN_4_^8−^, respectively. Cyanides can be easily produced from hydrocyanic acid, and there are several established synthesis routes for obtaining carbodiimides, e.g., through solid‐state metathesis or thermal decomposition of hydrogen cyanamide salts.^[^ ^23^ ^]^ The synthesis of compounds featuring CN_3_^5−^ directly from guanidine is challenging due to the basicity of amine and imide groups. Therefore, the synthesis of fully deprotonated guanidinate anions was achieved from elements with pressures above 32 GPa and a hydrogen‐free environment as reported for Sb(CN_3_)^[^ ^24^ ^]^ and Ln_3_O_2_(CN_3_) (Ln = La, Eu, Gd, Tb, Ho, Yb).^[^ ^25^ ^]^ The ortho‐nitridocarbonate anion CN_4_^8−^ remains unreported in its fully deprotonated form but is predicted in M_2_(CN_4_) stoichiometries with tetravalent cations.^[^ ^26^ ^]^ Increasing pressure above 70 GPa leads to the formation of *sp* ^3^‐hybridized carbon in the form of condensed CN_4_ tetrahedra, as reported for binary C─N compounds^[^ ^15^ ^]^ and lanthanoid polynitridocarbonates at megabar pressures.^[^ ^27^ ^]^ The addition of hydrogen in the system lowers the required pressure to 36 GPa for the synthesis of compounds featuring CN_4_ tetrahedra with *sp^3^*‐hybridized carbon as shown for *α*‐C(NH_2_) and *β*‐C(NH_2_).^[^ ^28^ ^]^

Guanidine (CN_3_H_5_) and its higher oligomers, melamine (C_3_N_6_H_6_) and melem (C_6_N_10_H_6_), are important precursors in the approach to synthesize graphitic carbon nitride (*g*‐C_3_N_4_).^[^ ^29^, ^30^, ^31^ ^]^ *g*‐C_3_N_4_ and its derivatives, e.g., poly(triazine imide) with intercalated LiCl, have promising mechanical, electrochemical, tribological, and photocatalytic properties.^[^ ^32^, ^33^ ^]^ The underlying polymerization mechanism for these compounds is based on classical thermal polycondensation under release of ammonia. We hypothesize that chemical and thermodynamic control of a solid‐state high‐pressure reaction may similarly lead to compounds featuring polymerized or oligomerized guanidinate fragments. In a high‐pressure synthesis of nitridocarbonates, the reaction can be controlled by the choice of the pressure regime and stoichiometry of the starting reaction mixture.^[^ ^15^, ^24^, ^27^ ^]^ In this work, we present several HPHT experiments for the synthesis of a novel compound, Bi_7_C_10_N_18_(N_3(1‐_ *_x_*_)_O_3_ *_x_*), which features 2D layers of deprotonated poly‐*N*‐(1,3,5‐triazin‐2‐*yl*)‐guanidine (Table 1).

**Table 1:** Summary of the experiments in DACs and their reaction conditions for the synthesis of Bi_7_C_10_N_18_(N_3(1‐_ *_x_*_)_O_3_ *_x_*) from elemental bismuth and either tetracyanoethylene (TCNE) or cyanuric triazide (CTA). For more details, see Supporting Information, Sec. A.

Experiment	Reagents	Pressure (GPa)
1^st^	Bi + TCNE	33.8(10)
2^nd^	Bi + CTA	38.1(10)–45.9(10)
3^rd^	Bi + CTA	50.1(10)

We conducted an LH‐DAC experiment, where elemental bismuth was embedded in tetracyanoethylene (C_6_N_4_), which served both as a reactant and a pressure‐transmitting medium. The DAC was loaded and sealed in a glovebox under an argon atmosphere, compressed to 30(1) GPa, and the Bi piece was laser‐heated to induce a reaction (highest temperature, *T *= 1600(300) K). The pressure increased to 33.8(10) GPa after the heating process. The reaction products were studied by means of synchrotron single‐crystal X‐ray diffraction (sc‐XRD) and powder X‐ray diffraction (PXRD) (see detailed description in Supporting Information, Sec. B). Sharp reflections appeared in the X‐ray diffraction images collected at the heated spot, which could not be explained by diffraction from *bcc*‐Bi. The diffraction pattern resulted from multiple single‐crystalline domains having a primitive trigonal lattice with lattice parameters *a, b * =  6.3338(7) Å and *c * =  19.497(2) Å. Initial structure solution and refinement resulted in a compound with the stoichiometry Bi_7_C_10_N_21_ (Figure 1, space group No. 163 *P* $\bar{3}$1*c*), which yielded an *R*_1_ of 4.3%, and a *wR*_2_ of 10.6% (see Table S1). The reconstructed reciprocal lattice planes of the phase are presented in Figure S2 and fulfill the systematic absences required for the space group *P* $\bar{3}$1*c*. After the structure was explored computationally in the framework of the density functional theory (DFT), the model was adapted to Bi_7_C_10_N_18_(N_3(1‐_ *_x_*_)_O_3_ *_x_*), as elaborated in the next section. Figure 2a depicts the azimuthally integrated XRD image of Bi_7_C_10_N_18_(N_3(1_ *_‐x_*_)_O_3_ *_x_*) at synthesis pressure, demonstrating that the proposed structure model accurately predicts the majority of observed peaks, particularly at lower 2*θ* values. The remaining peaks in the PXRD are attributed to the reflections from *bcc*‐Bi, for which equation of state (EoS) data were used to determine the pressure at the heated spot.^[^ ^34^ ^]^ The 2D‐PXRD map, which covered the heated spot, indicated the formation of Bi_7_C_10_N_18_(N_3(1‐_ *_x_*_)_O_3_ *_x_*) as the only crystalline product in addition to unreacted *bcc*‐Bi. The reaction took place at the edge of the bismuth piece, as visualized in Figure S3. After data acquisition, the DAC was decompressed to 26(3) GPa, and the reflections, which belonged to Bi_7_C_10_N_18_(N_3(1‐_ *_x_*_)_O_3_ *_x_*), disappeared from the XRD images. The synthesis was reproduced in two further experiments, where cyanuric triazide (C_3_N_12_) served as both reactant and pressure‐transmitting medium. The majority of the identified crystalline domains in the diffraction patterns belong to the same compound, Bi_7_C_10_N_18_(N_3(1‐_ *_x_*_)_O_3_ *_x_*).

**Figure 1:**
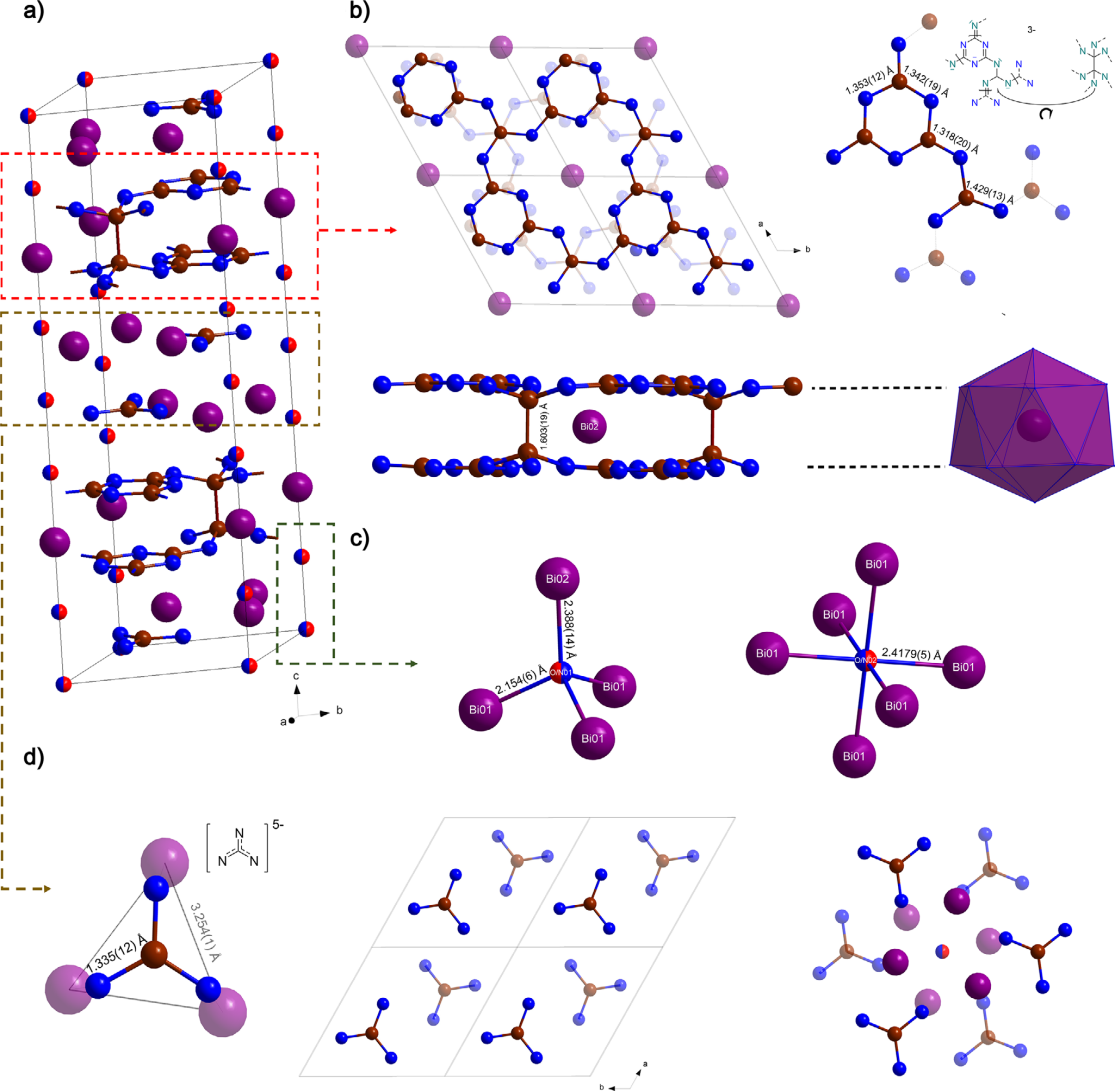
a) Unit cell of Bi_7_C_10_N_18_(N_3(1‐_ *_x_*_)_O_3_ *_x_*). b) Projection of the layered network [C_4_N_6_ *^x^* ^−^]*_n_* along the *a*‐axis, *c*‐axis, and the *N*‐(1,3,5‐triazin‐2‐*yl*)‐guanidine monomer with depicted bond lengths. The experimental interlayer C─C bond lengths are close to 1.6 Å, deviating from the ideal model of Bi_7_C_10_N_21_ extracted from DFT calculations. This deviation is due to reduction of carbon through the substitution of oxygen for the interstitial nitride position. c) Bismuth voids statistically occupied by O^2−^ and N^3−^ atoms. d) A guanidinate anion CN_3_^5−^ on top of a trimeric Bi01 unit and the quasi‐layered structure of CN_3_^5−^ anions along *c‐*axis. Bi, C, N, and O atoms are colored in purple, brown, blue, and red, respectively. Semi‐transparent atoms imply the next layer.

**Figure 2:**
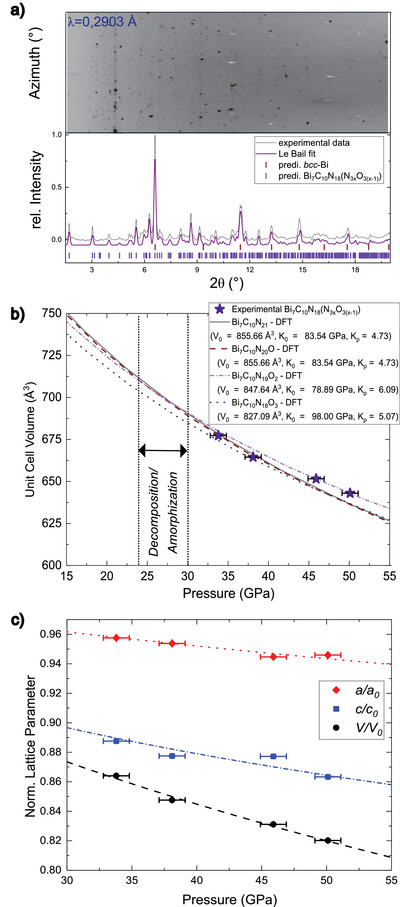
a) Azimuthal integrated X‐ray diffraction image, powder diffraction pattern, and Le Bail fit from polycrystalline Bi_7_C_10_N_18_(N_3(1‐_ *_x_*_)_O_3_ *_x_*) and unreacted *bcc*‐Bi at 33.8(10) GPa (1^st^ Experiment). b) Calculated equation of state (EoS) for all four structural models (Bi_7_C_10_N_21_, Bi_7_C_10_N_20_O, Bi_7_C_10_N_19_O_2_, and Bi_7_C_10_N_18_O_3_) compared to the experimental unit cell volumes of Bi_7_C_10_N_18_(N_3(1‐_ *_x_*_)_O_3_ *_x_*). c) Normalized lattice parameters plotted as function of pressure. Dashed line corresponds to the 2^nd^ order BM fit based on experimental values.

The following description of the structure model of Bi_7_C_10_N_18_(N_3(1‐_ *_x_*_)_O_3_ *_x_*) at 33.8(10) GPa is corroborated by DFT calculations. The experimental lattice parameters at 33.8(10) GPa show very close agreement with our DFT calculations performed for the idealized model Bi_7_C_10_N_21_ (no substitution of N^3‐^ by O^2‐^) by using approximations for the exchange‐correlation functionals (see Supporting Information, Sec. E, Table S7). In the following paragraphs, the bond length stated in brackets () after the experimental value corresponds to the calculated value. Bi_7_C_10_N_21_ can formally be split into fragments with 6 Bi^3+^ (Bi01, site 12*i*) (see Figure S1), 1 Bi^5+^ (Bi02, site 2*a*), 3 N^3−^ anions, 2 CN_3_^5−^ anions and two layers of condensed [C_4_N_6_^2−^]*_n_*. This is consistent with reported phases of binary bismuth nitrides and oxides, where bismuth exhibits +3^[^ ^18^, ^35^, ^36^ ^]^ and mixed +5/+3^[^ ^37^, ^38^ ^]^ oxidation states. The novelty of the structure lies in the formation of a quasi‐2D polymer, which is based on the *N*‐(1,3,5‐triazin‐2‐*yl*)‐guanidine monomer. The crystallographically distinguishable bond lengths in the *s‐*triazine unit are 1.353(12) Å (1.360 Å), 1.342(19) Å (1.326 Å), and 1.318(20) Å (1.343 Å) (see Figure 1b). In addition, the quasi‐layers of guanidinate CN_3_^5−^ anions (see Figure 1d) are of a similar arrangement to that observed in calcite structure types with a C─N bond length of 1.335(12) Å (1.333 Å) at 33.8(10) GPa. This bond length is close to those reported for Sb(CN_3_) (1.322(4) Å) at 32(1) GPa,^[^ ^24^ ^]^ indicating a bond order of 1.33. However, there is a slight discrepancy between the geometry‐optimized structure of Bi_7_C_10_N_21_ and the experimentally observed one. Structure refinement suggests that the 2D‐polymeric *N*‐(1,3,5‐triazin‐2‐*yl*)‐guanidine double layers are interconnected via a C─C lateral bond distance of 1.603(19) Å (Figures 1b and 3a), while the theoretical calculations for the idealized Bi_7_C_10_N_21_ model suggest that the layers must be ideally planar with no interlayer C─C bonding (see Figure 3a). The enthalpy difference from the geometry‐optimized Bi_7_C_10_N_21_ model (flat layers) to the experimental one of Bi_7_C_10_N_21_ (with C─C bonds, no geometry optimization) is in the range of 6 meV per atom (see Table S8), which can be exceeded by the available thermal energy at room temperature. However, we also considered the potential presence of minor oxygen impurities in the system, which often leads to the formation of oxonitrides.^[^ ^39^ ^]^

**Figure 3:**
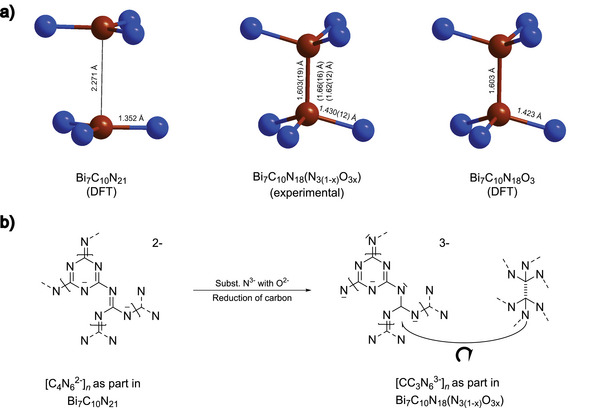
a) C─C distance of the guanidine‐derived unit of polymeric *N*‐(1,3,5‐triazin‐2‐*yl*)‐guanidine in the experimental crystal structures in comparison with the DFT‐calculated models of Bi_7_C_10_N_21_ and Bi_7_C_10_N_18_O_3_. b) Proposed mechanism for the formation of doubled layers of condensed *N*‐(1,3,5‐triazin‐2‐*yl*)‐guanidine by substituting interstitial N^3−^ by O^2−^.

First, significant oxygen impurities within the 2D polymer can be excluded due to the absence of unreasonable charges, deviating electron density (see Table S6), and bond lengths in the experimental and theoretical structure model. The bond distance for non‐polymerized guanidinate CN_3_^5−^ anions and the C─N bond in the triazine‐derived unit with the same bond order are longer than in typical carbonates CO_3_^2−^ (1.29 Å at ambient pressure)^[^ ^40^ ^]^ or the N─O bond length in calcite‐type nitrates NO_3_^−^ (1.25 Å at ambient pressure).^[^ ^41^ ^]^ In addition, the structure contains interstitial nitride atoms in tetrahedral and octahedral voids spanned by Bi atoms (Figure 1c). We systematically substitute these nitrogen sites in Bi_7_C_10_N_21_ by oxygen in our theoretical calculations, using PBEsol+D3 functionals that resulted in the three additional structure models Bi_7_C_10_N_20_O_1_, Bi_7_C_10_N_19_O_2_, and Bi_7_C_10_N_18_O_3_. Substituting nitrogen at position N01 by oxygen (Site 4e, tetrahedral void, Bi_7_C_10_N_19_O_2_) does not affect the lateral C─C bonding (2.33 Å) situation of the polymer in the geometry‐optimized structure obtained by DFT calculations. However, replacing N02 (Site 2b, octahedral void, Bi_7_C_10_N_20_O + Site 2*b *+ 4*e*, Bi_7_C_10_N_18_O_3_) with oxygen accurately reproduces the formation of C─C single bonds close to 1.6 Å, which is in accordance with our experimental structure. Additionally, the enthalpy difference from the idealized Bi_7_C_10_N_21_ model to the oxygen‐substituted phases is in the range of 13 meV per atom (see Figure S4), which can easily be exceeded by the available thermal energy at room temperature, thereby making all four models possible.

The formation of the C─C single bond in the case of Bi_7_C_10_N_18_O_3_ and Bi_7_C_10_N_20_O is supported by our DFT‐calculated charge density map and electron localization function through the C─C lateral plane (see Figure S5–S7). We also note that the oxygen substitution both at the tetrahedral void and the octahedral void retains the space group symmetry of *P* $\bar{3}$1*c* (No. 163). Compared to Bi_7_C_10_N_21_ with flat 2D layers, the Bader charge analysis of Bi_7_C_10_N_18_O_3_ has shown that the substitution of N^3−^ to O^2−^ has caused a substantial reduction in the formal charges at the C (4*f*) Wyckoff site (see Figure 3, Table S9), arguing for carbon closer to oxidation state +3. Substitution of oxygen by nitrogen in our structural models barely affects the refinement paraments *ΔR_1_* (<0.01%) and *ΔwR_2_* (<0.2%) due to the dominant contribution of bismuth atoms to the structure factors. Additionally, the combination of reduced dataset completeness caused by the opening angle of the diamond anvils and the possible variations of oxygen content in individual crystal domains (see Table S6) means we are not able to precisely determine the final stoichiometry in the experimental data. Therefore, we opted for the experimental model, where the tetrahedral and octahedral bismuth voids are randomly occupied by oxygen and nitrogen, to Bi_7_C_10_N_18_(N_3(1‐_ *_x_*_)_O_3_ *_x_*) with *x* = [0,1]. Considering that the calculated models Bi_7_C_10_N_20_O and Bi_7_C_10_N_18_O_3_ are the closest to the experimental findings, we estimate *x* close to 2/3.

The DFT‐calculated lattice parameters were used to derive a 3^rd^ order Birch–Murnaghan (BM) EoS (see Figure 2b,c).^[^ ^42^, ^43^ ^]^ The pressure‐volume dependence obtained from our three independent experiments is in good agreement with the calculated EoS, well within the experimental pressure uncertainty of 1 GPa. The resulting bulk moduli (*K*_0_) of 82–98 GPa from the calculated EoS fits are lower than that of our previously synthesized calcite‐type SbCN_3_ (*K*_0 _= 214 GPa)^[^ ^24^ ^]^ and calcite‐type carbonates (*K*_0 _≈ 100 GPa).^[^ ^44^ ^]^ When considering the normalized lattice parameters (Figure 2c), the *c*‐axis of the unit cell appears to be more compressible than the in‐plane (*a,b)*‐axes, largely contributing to the overall compressibility of Bi_7_C_10_N_18_(N_3(1‐_ *_x_*_)_O_3_ *_x_*). The 2D polymeric network of *N*‐(1,3,5‐triazin‐2‐*yl*)‐guanidine and the quasi layers of CN_3_^5−^ anions expand along the (*a,b)*‐axes, where the covalent C─N bonds are less compressible than the interlayer distances along the *c*‐axis, which mainly rely on weaker van der Waals (vdW) and ionic interactions between the quasi‐layers.

DFT calculations were performed to further investigate the lattice stability and electronic structure of suggested structural models. The calculated phonon dispersion relations show that the crystal structures of all four structural models are dynamically stable at the synthesis pressure of 34 GPa and consistent with our experimental observations (Figures 4a,b and S8).

**Figure 4:**
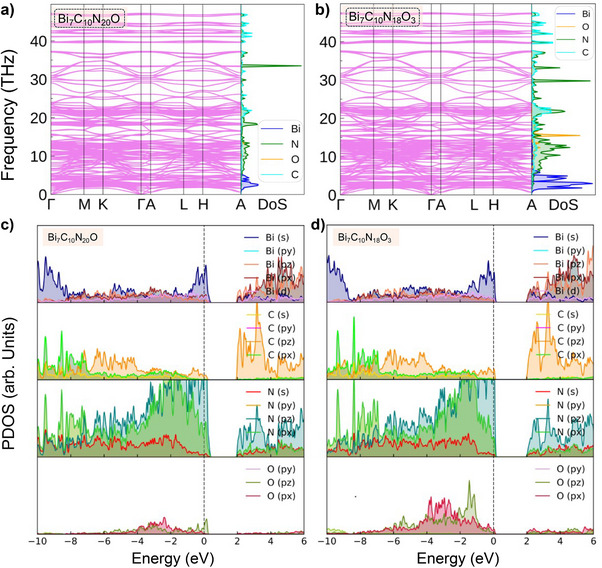
Phonon dispersion for a) Bi_7_C_10_N_20_O and b) Bi_7_C_10_N_18_O_3_ at a synthesis pressure of 34 GPa. All calculated vibrational frequencies are real, indicating lattice dynamical stability. Projected electronic density of states (PDOS) for c) Bi_7_C_10_N_20_O and d) Bi_7_C_10_N_18_O_3_ at 34 GPa.

The electronic density of states (DOS) for Bi_7_C_10_N_21_ is primarily dominated by nitrogen *p*‐orbitals, and the corresponding N *p*‐orbital resolved DOS reveals that the N *p_z_‐*orbitals make up the majority of the contribution to the electronic density near the Fermi energy together with the C *p_z_‐*orbitals, supporting the model of a delocalized π‐electron network from *N*‐(1,3,5‐triazin‐2‐*yl*)‐guanidine (see Figures S9 and S10). However, oxygen substitution and the formation of the quasi‐2D double layers cause the formation of a pseudo gap just above the Fermi level, supporting the idea of π‐bonds transforming into C─C single bonds of the guanidine‐derived unit in *N*‐(1,3,5‐triazin‐2‐*yl*)‐guanidine (for Bi_7_C_10_N_20_O and Bi_7_C_10_N_18_O_3_, see Figure 4c,d). Both Bi_7_C_10_N_20_O and Bi_7_C_10_N_18_O_3_ structural models are preserving the metallic character (see Figure S11), and no Raman signal was experimentally observed.

Moreover, phonon calculations at lower pressures of 30 and 20 GPa reveal lattice instability for both end members, Bi_7_C_10_N_21_ and Bi_7_C_10_N_18_O_3_, indicated by the appearance of imaginary phonon branches in the phonon dispersion (see Figure S12). This dynamic instability is arising from a weaker bonding between the Bi02 (Site 2*a*) and N/O01 (Site 4*e*, tetrahedral void) atoms when the pressure within the cell gets reduced during the decompression. Besides, we observed the disappearance of crystallinity at 26(3) GPa in the XRD pattern as pointed out earlier.

In conclusion, the validation of the structure model Bi_7_C_10_N_18_(N_3(1‐_ *_x_*_)_O_3_ *_x_*) is supported by reasonable bond lengths, reproducibility, and by our DFT calculations. Interstitial N^3‐^ and O^2‐^ anions and the lower cation charge of bismuth tend to cause condensation of guanidinate anions to compensate for the less available electrons that would be required to stabilize CN_3_^5−^. Following this example for synthesis design, we can envision lower cation charges, and the variation of the cation radius might lead to other oligomers and polymers of *sp* ^2^‐hybridized carbon nitride networks approaching *g*‐C_3_N_4_. Our theoretical investigations revealed that the reason for dynamical instability in Bi_7_C_10_N_18_(N_3(1‐_ *_x_*_)_O_3_ *_x_*) under decompression is caused by the highly coordinated Bi02 species. A rational design to reproduce this anionic poly‐*N*‐(1,3,5‐triazin‐2‐*yl*)‐guanidine network, with the potential of recoverability to ambient pressure, might be the choice of a divalent cation for stabilization. The establishment of an inorganic nitridocarbonate family featuring *sp^2^*‐hybridized carbon can compete with classical polycondensation reactions, and the synthesis of Bi_7_C_10_N_18_(N_3(1‐_ *_x_*_)_O_3_ *_x_*) proves the concept that HPHT conditions, driven by thermodynamics, can access 2D materials with high polymerization degrees.

Additional references are cited in the Supporting Information.^[^ ^45^, ^46^, ^47^, ^48^, ^49^, ^50^, ^51^, ^52^, ^53^, ^54^, ^55^, ^56^, ^57^, ^58^, ^59^, ^60^, ^61^ ^]^ Experimental structural data has been deposited at the Cambridge Crystallographic Data Centre (CCDC) under the depository number 2428895.^[^ ^62^ ^]^

## Conflict of Interests

The authors declare no conflict of interest.

## Supporting information



Supporting Information

Supporting Information

## Data Availability

The data that support the findings of this study are available from the corresponding author upon reasonable request.
