# Supplementary material for: High‐Pressure Synthesis of Crystalline Double‐Layer Carbon Nitride Networks Stabilized in Bi7C10N18(N3(1‐ x )O3 x )
Source: Angew Chem Int Ed Engl. 2025 Jul 23;64(35):e202506406. doi: 10.1002/anie.202506406 (PMC12377433; doi:10.1002/anie.202506406)
Supplement: Supplementary file 1 — Supporting Information [file ANIE-64-e202506406-s001.pdf]

Supporting Information:

High-Pressure Synthesis of Crystalline Double-Layer Carbon Nitride Networks Stabilized in $Bi_7C_{10}N_{18}(N_{3(1-x)}O_{3x})$

Lukas Brüning[a]*, Nityasagar Jena[b], Pascal L. Jurzick[a,c], Elena Bykova[d], Nico Giordano[e], Mohamed Mezouar[f], Igor A. Abrikosov[b], Maxim Bykov[a]*

[a] L. Brüning*, P.L Jurzick, Prof. Dr. M. Bykov*
Institute for Inorganic and Analytical Chemistry, Goethe University Frankfurt
60438, Frankfurt am Main, Germany
E-Mail: l.bruening@chemie.uni-frankfurt.de, maxim.bykov@chemie.uni-frankfurt.de

[b] Dr. N. Jena, Prof. I. A. Abrikosov
Department of Physics, Chemistry and Biology (IFM), Linköping University
SE-58183, Linköping, Sweden

[c] P. L. Jurzick
Institute for Inorganic Chemistry, University of Cologne
50939, Cologne, Germany

[d] Prof. Dr. E. Bykova
Institute of Geoscience, Goethe University Frankfurt
60438, Frankfurt am Main, Germany

[e] Dr. N. Giordano
Deutsches Elektronen-Synchrotron (DESY),
22607, Hamburg, Germany

[f] Dr. M. Mezouar
European Synchrotron Radiation Facility (ESRF),
38043, Grenoble, France

Contents

Section A: Synthesis 2
Section B: X-Ray Diffraction studies and further Programs 3
Section C: SC-XRD Data of $Bi_7C_{10}N_{18}(N_{3(1-x)}O_{3x})$, ... 4
Section D: 2D-PXRD Map 8
Section E: DFT Calculations 9

Section A: Synthesis

Three independent experiments were performed for the synthesis of $Bi_7C_{10}N_{18}(N_{3(1-x)}O_{3x})$, using BX90 diamond anvil cells with Boehler-Almax type diamonds (200 µm culet diameter and 60° opening angle).

In the 1st experiment, tetracyanoethylene (C_6N_4) served as reactant and pressure transmitting medium (PTM). A piece of bismuth with a diameter of 40 um was placed in the 100 µm hole of the Re gasket, which had been preindented to a thickness of 24(4) µm. The sample hole was filled with C_6N_4 under argon atmosphere and compressed to 32(1) GPa. The bismuth piece was laser heated on both sides with a Nd:YAG laser (highest temperature, T=1600(300) K) until a reaction was observed by XRD. After heating, the pressure increased to 33.8(10) GPa and a single-crystal structure refinement revealed the presence of $Bi_7C_{10}N_{18}(N_{3(1-x)}O_{3x})$. Unreacted *bcc*-Bi was present in the PXRD pattern (see Figure 2(a) of the main text). The DAC was decompressed to 26(3) GPa and the $Bi_7C_{10}N_{18}(N_{3(1-x)}O_{3x})$ phase was no longer present in the PXRD patterns, which indicates phase decomposition.

In the 2nd experiment, cyanuric triazide (C_3N_{12}) served as reactant and PTM. A piece of bismuth with a diameter of 35 µm was placed in the 100 µm hole of the Re gasket, which was preindented to a thickness of 24(4) µm. The sample hole was filled with C_3N_{12} and compressed to 30(1) GPa. The bismuth piece was laser-heated on both sides with a Nd:YAG laser (no temperature measurement, T>1000 K) until a reaction was observed by XRD. After heating, the pressure increased to 38.1(10) GPa and $Bi_7C_{10}N_{18}(N_{3(1-x)}O_{3x})$ was solved from single-crystal data. In a second heating process, the spot was heated again and lead to a pressure increase to 45.9(10) GPa. At this pressure, the $Bi_7C_{10}N_{18}(N_{3(1-x)}O_{3x})$ phase recrystalized.

In the 3rd experiment, Cyanuric triazide (C_3N_{12}) served as reactant and PTM. A piece of bismuth with a diameter of 30 µm was placed in the 100 µm hole of the Re gasket, which was preindented to a thickness of 24(4) µm. The sample hole was filled with C_3N_{12} and compressed to 48(1) GPa. The bismuth piece was laser-heated at one side with a Nd:YAG laser (no temperature measurement, T>1000 K). After the heating process, the pressure increased to 50.1(10) GPa and $Bi_7C_{10}N_{18}(N_{3(1-x)}O_{3x})$ was solved from single-crystal data. IR-spectra of the precursor C_3N_{12} indicated slight impurities of oxygen.

The pressure in all experiments was determined using the EoS of unreacted and recrystallized *bcc*-Bi at the spot of measurement.[33]

Section B: X-Ray Diffraction studies and further Programs

The LH-DAC experiments including the reaction products were studied by means of single-crystal and powder X-ray diffraction on the synchrotron beamline P02.2 at DESY in Hamburg (λ≈0.2903 Å, Perkin Elmer XRD1621 flat panel detector) [45,46] and on the synchrotron beamline ID27 (λ≈0.3738 Å, Eiger2 X 9M CdTe flat panel detector) at ESRF in Grenoble.[47] The beam diameters were about 0.7 µm(ID27) and 3 µm (DESY).

At synthesis pressure and selected pressure points during decompression, the sample areas were scanned with the X-ray beam, resulting in a 2D-PXRD grid. At selected points of the grid, we collected single-crystal data with a narrow 0.5° scanning step from -30° to +30° rotation angle ω.

Diffraction data analysis up to reflection data file of the multi domain measurements was performed with CrysAlisPro software and the integrated Domain Auto Finder (DAFi) program[40]. The crystal structures were solved and refined based on single-crystal diffraction data using the program OLEX2.[49] Within the OLEX2 software interface, ShelXT was used for generating a suitable structure model based on output of the integration and ShelXL was used for least squares refinement of the structure model. At some points of the grid, the raw image was integrated azimuthally to obtain a powder pattern using Dioptas.[50] Le Bail fits of $Bi_7C_{10}N_{18}(N_{3(1-x)}O_{3x})$ were performed using JANA2006.[51] The structures were visualized with Diamond 5.02. Polynator[52] was used to calculate the deviation between occurring polyhedra in experimental crystal structures and well-defined geometrical bodies.

Section C: SC-XRD Data of $Bi_7C_{10}N_{18}(N_{3(1-x)}O_{3x})$

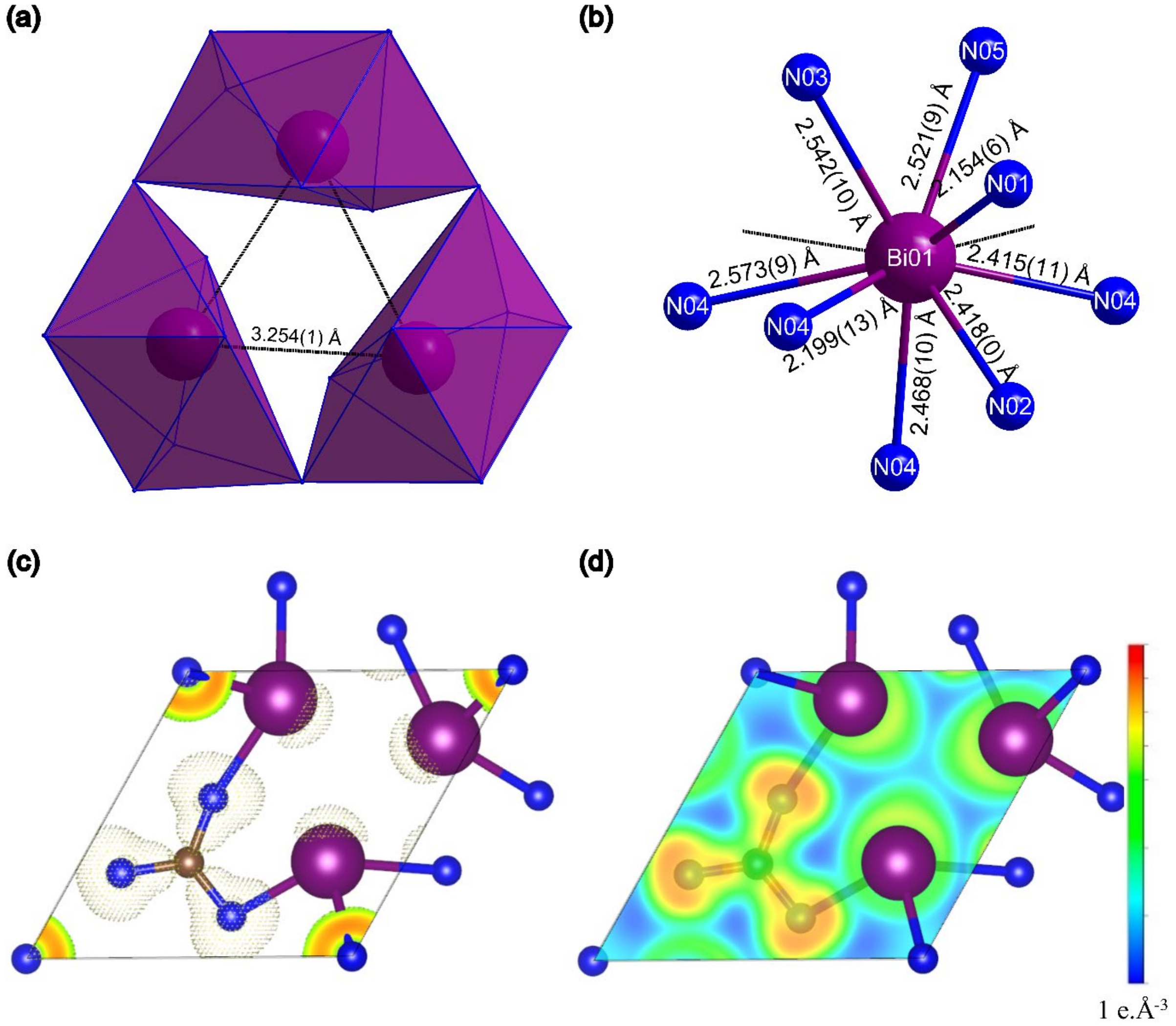

Figure S1: (a) Trimeric unit of Bi01 atoms (model $Bi_7C_{10}N_{21}$), featuring space for bismuth lone pairs. **(b)** Bi01 atom with depicted bond lengths to neighboring atoms. **(c)** The Electron localization function (ELF) isosurfaces of the trimer with a level of 0.6 e/Å^3 and **(d)** Section of the ELF along the plane spanned by the Bi01 trimer. For more details, see section E and Figures S5-7. Bi and N atoms are colored in purple and blue respectively.

Table S1: Experimental single-crystal structure details of $Bi_7C_{10}N_{18}(N_{3(1-x)}O_{3x})$ structure solutions. Bismuth atoms were refined with anisotropic displacement parameters. For the best refinement we opted the model to $Bi_7C_{10}N_{19}O_2$ (see main text, site 4*e* N01/O01 with 50% occupancy for N and O, site 2*b* fully occupied by O). Since no significant differences are observed by substituting O with N in the refinement parameters, all refinements of supporting data sets were kept to $Bi_7C_{10}N_{21}$ for simplicity.

Experiment	1st	2nd	2nd	3rd
Chemical Formula	$Bi_7C_{10}N_{19}O_2$ ($Bi_7C_{10}N_{21}$)	$Bi_7C_{10}N_{21}$	$Bi_7C_{10}N_{21}$	$Bi_7C_{10}N_{21}$
Pressure (GPa)	33.8(10)	38.1(10)	45.9(10)	50.1(10)
Beamline	P02.2 (DESY)	P02.2 (DESY)	P02.2 (DESY)	ID27 (ESRF)
Wavelength (Å)	0.2903	0.2903	0.2903	0.3738
Crystal sytem, space group	Trigonal, *P*-31*c* (No. 163)	Trigonal, *P*-31*c* (No. 163)	Trigonal, *P*-31*c* (No. 163)	Trigonal, *P*-31*c* (No. 163)
a, *c* (Å)	6.3338(7), 19.495(2)	6.3091(8), 19.276(9)	6.2486(16), 19.270(6)	6.257(3), 18.965(11)
V ($Å^3$)	677.31(17)	664.5(3)	651.6(4)	643.0(7)
Z	2	2	2	2
No. of measured, independent and observed [$I > 2\sigma(I)$] reflections	2593, 1262, 876	2372, 930, 499	839, 367, 252	868, 489, 404
R_{int}	0.036	0.077	0.068	0.035
$R[F^2 > 2\sigma(F^2)], wR(F^2), S$	0.043, 0.106, 1.01 (0.043, 0.106, 1.01)	0.066, 0.192, 1.06	0.090, 0.236, 1.13	0.089, 0.268, 1.14
No. Of parameters	36	36	36	36
$\Delta\rho max, \Delta\rho min$ (e $Å^{-3}$)	3.34, −3.46 (3.34, −3.46)	8.77, −4.29	4.64, −3.94	5.21, −5.01

Best Dataset (1st Experiment):

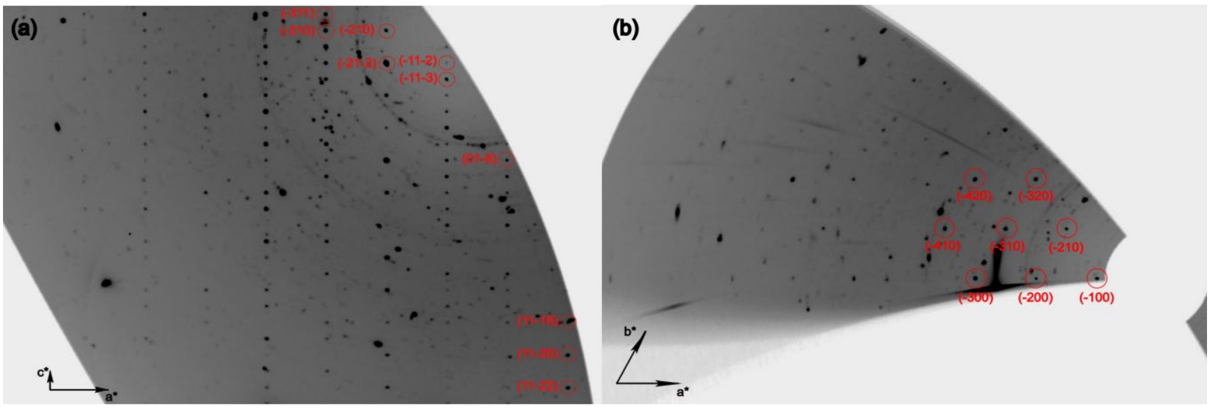

Figure S2: (a) (*h1l*)- and **(b)** (*hk0*)-reciprocal lattice plane of $Bi_7C_{10}N_{18}(N_{3(1-x)}O_{3x})$ at 33.8(10) GPa (1st experiment) with indexed reflections. The indexed reflections in **(a)** fulfil the condition $hh\overline{2h}l$; $l = 2n$ for a *c*-glide plane perpendicular to the [1-10] direction in the hexagonal unit cell setting for primitive trigonal space groups. To convert *hkl* to *hkil*, $i = -(h + k)$ holds.

Table S2: Fractional coordinates and Wyckoff sites of $Bi_7C_{10}N_{18}(N_{3(1-x)}O_{3x})$ at 33.8(10) GPa.

Atom	Wyck.	x/a	y/b	z/c	$U_{eq.}$ (Å^2)
Bi01	12i	0.66394(7)	0.75430(7)	0.42381(2)	0.00935(10)
Bi02	2a	0	0	1/4	0.0379(4)
N/O01	4e	0	0	0.3725(7)	0.0051(19)
N/O02	2b	0	0	1/2	0.003(2)
N03	12i	0.7315(17)	0.5748(16)	0.3149(4)	0.0072(13)
N04	12i	0.8570(16)	0.5602(16)	0.4530(4)	0.0072(13)
N05	12i	1.1262(17)	0.6855(17)	0.3111(4)	0.0074(13)
C01	4f	1/3	2/3	0.2911(7)	0.005(2)
C02	12i	0.9995(19)	0.503(2)	0.3141(5)	0.0076(14)
C03	4f	2/3	1/3	0.4522(9)	0.010(3)

Supporting Datasets:

Table S3: Fractional coordinates and Wyckoff site of $Bi_7C_{10}N_{18}(N_{3(1-x)}O_{3x})$ at 38.1(10) GPa (2nd Experiment).

Atom	Wyck.	x/a	y/b	z/c	$U_{eq.}$(Å^2)
Bi01	12i	0.33746(13)	0.24793(12)	0.42563(6)	0.0192(4)
Bi02	2a	0	0	1/4	0.088(2)
N/O01	4e	0	0	0.3735(16)	0.001(3)
N/O02	2b	0	0	1/2	0.014(7)
N03	12i	0.158(3)	-0.268(3)	0.3167(15)	0.016(3)
N04	12i	0.138(3)	0.441(3)	0.4525(14)	0.013(3)
N05	12i	0.433(3)	0.124(3)	0.3118(13)	0.014(3)
C01	4f	2/3	1/3	0.292(3)	0.025(8)
C02	12i	0.391(4)	-0.099(4)	0.3151(18)	0.018(4)
C03	4f	1/3	2/3	0.452(3)	0.015(6)

Table S4: Fractional coordinates and Wyckoff site of $Bi_7C_{10}N_{18}(N_{3(1-x)}O_{3x})$ at 45.9(10) GPa (2nd Experiment).

Atom	Wyck.	x/a	y/b	z/c	$U_{eq.}$(Å^2)
Bi01	12i	0.6615(4)	0.7545(4)	0.57468(9)	0.0174(8)
Bi02	2a	1	1	1/4	0.066(3)
N/O01	4e	1	1	0.620(3)	0.001(11)
N/O02	2b	1	1	1/2	0.001(16)
N03	12i	0.717(12)	0.581(13)	0.685(3)	0.035(14)
N04	12i	0.846(7)	0.569(7)	0.5482(16)	-0.001(6)
N05	12i	1.116(9)	0.685(9)	0.689(2)	0.016(9)
C01	4f	2/3	1/3	0.707(3)	0.014(17)
C02	12i	0.902(9)	0.520(9)	0.686(2)	0.007(9)
C03	4f	1/3	2/3	0.6956(4)	0.03(2)

Table S5: Fractional coordinates and Wyckoff site of $Bi_7C_{10}N_{18}(N_{3(1-x)}O_{3x})$ at 50.1(10) GPa (3rd Experiment). C01-C01 distance is fixed to 1.6 Å in this refinement.

Atom	Wyck.	x/a	y/b	z/c	$U_{eq.}$(Å^2)
Bi01	12i	0.33918(19)	0.2454(2)	0.57549(7)	0.0242(6)
Bi02	2a	0	0	1/4	0.064(2)
N/O01	4e	0	0	0.628(3)	0.022(9)
N/O02	2b	0	0	1/2	0.020(12)
N03	12i	0.153(6)	-0.270(6)	0.6851(18)	0.030(6)
N04	12i	0.137(5)	0.435(6)	0.5493(17)	0.025(6)
N05	12i	0.435(4)	0.125(4)	0.6930(14)	0.014(4)
C01	4f	2/3	1/3	0.7079(4)	0.034(14)
C02	12i	0.108(8)	-0.506(7)	0.684(2)	0.032(8)
C03	4f	1/3	2/3	0.550(2)	0.009(7)

Table S6: Analysis of the two best single-crystal refinements of $Bi_7C_{10}N_{18}(N_{3(1-x)}O_{3x})$ by refining the occupancy of individual Wyckoff sites free, while leaving the other Wyckoff sites fully occupied.

Site	1st Exp. 34(1) GPa free occ. N	2nd Exp. 38(1) GPa free occ. N	1st Exp. 34(1) GPa free occ. O	2nd Exp. 38(1) GPa free occ. O	1st Exp. 34(1) GPa free occ. Bi	2nd Exp. 38(1) GPa free occ. Bi
N/O01 (tetrahdral void, site 4*e*)	1.40(9)	1.2(2)	1.10(7)	0.79(10)	-	-
N/O02 (octahedral void, site 2*b*)	1.30(11)	1.0(2)	1.02(9)	0.93(16)	-	-
N03	1.04(5)	0.97(8)	0.80(4)	0.79(8)	-	-
N04	1.02(5)	1.02(10)	0.81(4)	0.76(7)	-	-
N05	1.08(5)	1.00(9)	0.85(4)	0.77(7)	-	-
Bi01	-	-	-	-	0.95(2)	1.02(4)
Bi02	-	-	-	-	0.95(2)	1.08(4)

Section D: 2D-PXRD Map

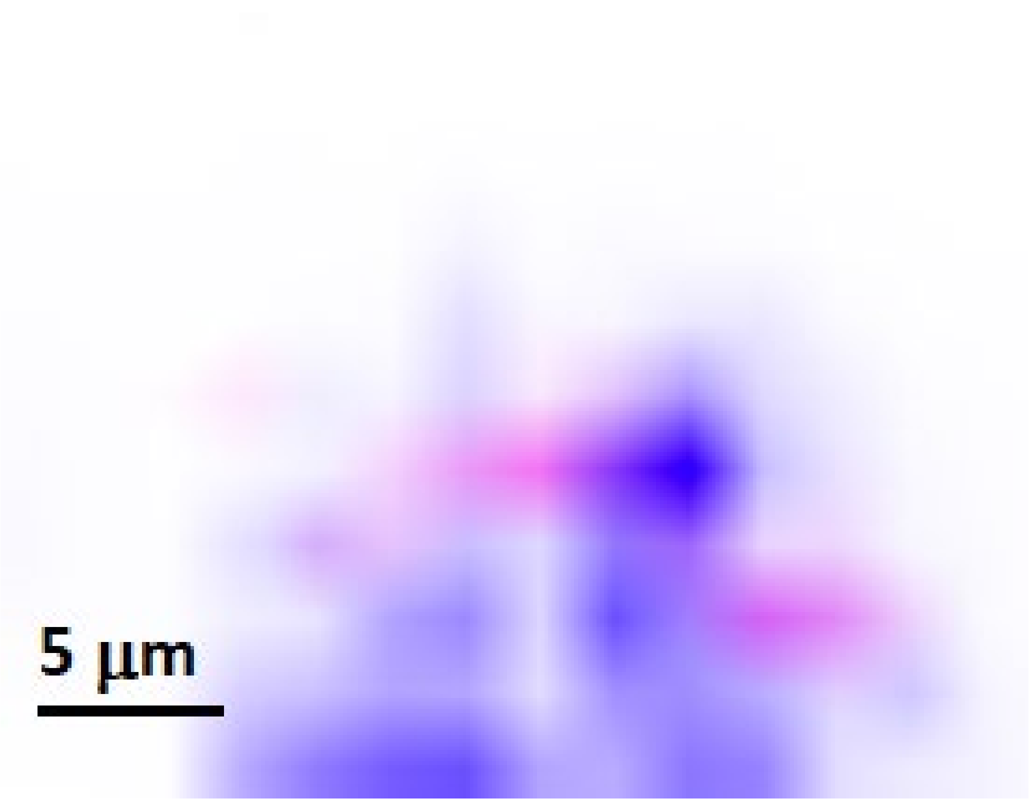

Figure S3: 2D-PXRD map after synthesis (1st experiment, 33.8(10) GPa). Blue color code corresponds to the intensity of the (110) reflection of *bcc*-Bi and purple correspond to the intensity of the (100) reflection of $Bi_7C_{10}N_{18}(N_{3(1-x)}O_{3x})$.

Section E: DFT Calculations

First-principles calculations based on the framework of density functional theory (DFT) were performed using the Projector-Augmented-Wave (PAW) method[53,54], as implemented in the plane wave code Vienna Ab initio Simulation Package (VASP), version 6.4.3[55,56]. The exchange-correlation functionals were treated using the generalized gradient approximation (GGA) in the form of the Perdew–Burke–Ernzerhof (PBE) functional [56] as well as using PBEsol functional[49]. The equilibrium lattice constants were determined using the PBE and PBEsol functionals in conjunction with the empirical dispersion correction, DFT-D3, incorporating the Becke-Johnson (B-J) damping function[58], within the Kohn−Sham framework for the total energy calculations[58]. Our calculated lattice parameters using the PBEsol+D3(B-J) approach exhibited close agreement with the experimental pressure-volume data (Table S6). Therefore, this combination of functionals was adopted for subsequent material property calculations.

The PAW potentials were sourced from the VASP PBE potential library, with valence electron configurations of $5d^{10}\,6s^2\,6p^3$ for Bi (15 valence electrons), $2s^2\,2p^2$ for C (4 valence electrons), $2s^2\,2p^3$ for N (5 valence electrons), and $2s^2\,2p^4$ for O (6 valence electrons). A plane-wave kinetic energy cutoff of 900 eV was set for the self-consistent energy convergence with an energy tolerance of 10^{-8} eV. Variable cell relaxations, including lattice parameters and atomic positions were carried out using the conjugate-gradient (CG) optimization scheme until the residual atomic forces were below 10^{-3} eV/Å and the Pullay stress was reduced to < 0.01 GPa. For the Brillouin zone (BZ) sampling a Γ-centered k-mesh was used on a 12x12x3 k-point grid.

The equations-of-state (EoS) for $Bi_7C_{10}N_{21}$, $Bi_7C_{10}N_{20}O$, $Bi_7C_{10}N_{19}O_2$, and $Bi_7C_{10}N_{18}O_3$ were determined by performing structural relaxations at various scaled volumes relative to the equilibrium ground state geometry obtained at 34 GPa. The resulting pressure-volume data were fitted to a 3rd-order Birch–Murnaghan equation to extract the fitting parameters, including the equilibrium volume, bulk modulus, and its pressure derivative.

The ground-state electron density and electronic density of states (EDOS) were computed using the same 12x12x3 k-mesh, employing the tetrahedron smearing method with Blöch corrections for atom- and orbital-projected EDOS calculations. The electron localization functions (ELF) were also computed using the same grid density. Crystal structure visualization, ELF distribution map, and the charge density isosurfaces were rendered using the VESTA visualization software.[60]

The phonon dispersion relations at a lower synthesis pressure of 34 GPa, where the $Bi_7C_{10}N_{21}$ phase has been experimentally observed, were calculated in the harmonic approximation using the finite displacement method as implemented in Phonopy package.[61] The interatomic forces for several symmetry-imposed displacement geometries within a supercell of 3x3x2 were evaluated through single-point energy minimization in VASP. The second-order interatomic force constants (IFCs) were processed in Phonopy to obtain the phonon dispersion relations and the corresponding phonon density of states (PhDOS). The same computational setup was employed to compute the lattice stability of several oxygen substituted phases, such as $Bi_7C_{10}N_{20}O$, $Bi_7C_{10}N_{19}O_2$, and $Bi_7C_{10}N_{18}O_3$ at a calculation pressure of 34 GPa. Atomic charge transfer analysis was conducted using the Bader's charge density partitioning method, incorporating both the core and valence charge densities into the charge density partitioning scheme that accurately accounts for charge transfer between different elemental species and atomic sites.[62]

Table S7: Experimental lattice parameters of hP76-$Bi_7C_{10}N_{21}$ ($P\bar{3}1c$, space group (S.G.) number #163) at a synthesis pressure of 34 GPa, and the DFT calculated lattice parameters at 34 GPa using different exchange-correlation functionals. The DFT calculations using PBEsol+D3 (B-J) are in better agreement with the experimental lattice parameters in comparison with results obtained with PBE+D3 functional. The former approximation was therefore adopted for subsequent material property calculations.

	a,b **(Å)**	***c*** **(Å)**	**V (Å^3)**	**Density (g/cm^3)**
Experimental	6.334	19.495	677.30	9.204
PBE+D3	6.403	19.556	694.43	8.977
PBEsol+D3	6.372	19.233	676.36	9.217

Table S8: Experimental lattice parameter at a synthesis pressure of 34 GPa, and the DFT calculated lattice parameters of pure $Bi_7C_{10}N_{21}$, and various oxygen substituted phases at 34 GPa using different oxygen concentration around the Bi02 Wyckoff position.

**Compounds (at 34 GPa)**	***a, b*** **(Å)**	***c*** **(Å)**	***c/a***	***V*** **(Å^3)**	**Density (g/cm^3)**	**Sym. (S.G.)**	**Enthalpy (eV/atom)**	**C-C bond (Å)**
**DFT:**								
$Bi_7C_{10}N_{21}$	6.372	19.233	3.018	676.363	9.217	*P*-31*c* (No. 163)	-5.415	2.271
$Bi_7C_{10}N_{20}O$ (O at site 2*b*)	6.355	19.092	3.004	667.720	9.346	*P*-31*c* (No. 163)	-5.43	1.574
$Bi_7C_{10}N_{19}O_2$ (O at site 4*e*)	6.343	19.487	3.072	679.027	9.2	*P*-31*c* (No. 163)	-5.473	2.337
$Bi_7C_{10}N_{18}O_3$ (O at site 2*b*+4*e*)	6.339	19.177	3.025	667.378	9.371	*P*-31*c* (No. 163)	-5.483	1.603
**Experiment:**								
$Bi_7C_{10}N_{18}(N_{3(1-x)}O_{3x})$	6.334	19.495	3.077	677.302	9.204	*P*-31*c* (No. 163)	--	1.602

Table S9: Bader charge transfer between different elements and sublattice positions (Wyckoff positions) for $Bi_7C_{10}N_{21}$, $Bi_7C_{10}N_{20}O$, $Bi_7C_{10}N_{19}O_2$, and $Bi_7C_{10}N_{18}O_3$ calculated at a pressure of 34 GPa. The + sign refers to loss of charge and the – sign refers to the gain in charge by the elements at different Wyckoff positions.

$Bi_7C_{10}N_{21}$ (34 GPa)	Wyckoff positions	Bader charge	$Bi_7C_{10}N_{20}O$ (34 GPa)	Wyckoff positions	Bader charge	$Bi_7C_{10}N_{19}O_2$ (34 GPa)	Wyckoff positions	Bader charge	$Bi_7C_{10}N_{18}O_3$ (34 GPa)	Wyckoff positions	Bader charge
Bi (14-atoms)	(12i)	1.92	Bi (14-atoms)	(12i)	1.89	Bi (14-atoms)	(12i)	1.75	Bi (14-atoms)	(12i)	1.87
	(2a)	2.35		(2a)	2.21		(2a)	2.41		(2a)	2.37
N(42-atoms)	(4e)	-1.37	O (2-atoms)	**(2b)**	-1.28	O (4-atoms)	(4e)	-1.27	O (6-atoms)	(4e)	-1.17
	(2b)	-1.32	N (40-atoms)	(4e)	-1.23	N (38-atoms)	(2b)	-1.26		**(2b)**	-1.05
	(12i)	-0.69		(12i)	-0.76		(12i)	-0.7	N (36-atoms)	(12i)	-0.62
	(12i)	-1.15		(12i)	-1.06		(12i)	**-0.88**		(12i)	-1.07
	(12i)	-0.81		(12i)	-0.79		(12i)	-0.78		(12i)	-0.83
C(20-atoms)	(4f)	0.59	C (20-atoms)	(4f)	0.6	C (20-atoms)	(4f)	0.62	C (20-atoms)	(4f)	0.58
	(12i)	0.71		(12i)	0.66		(12i)	0.72		(12i)	0.55
	(4f)	0.41		(4f)	0.4		(4f)	**-0.16**		(4f)	0.31

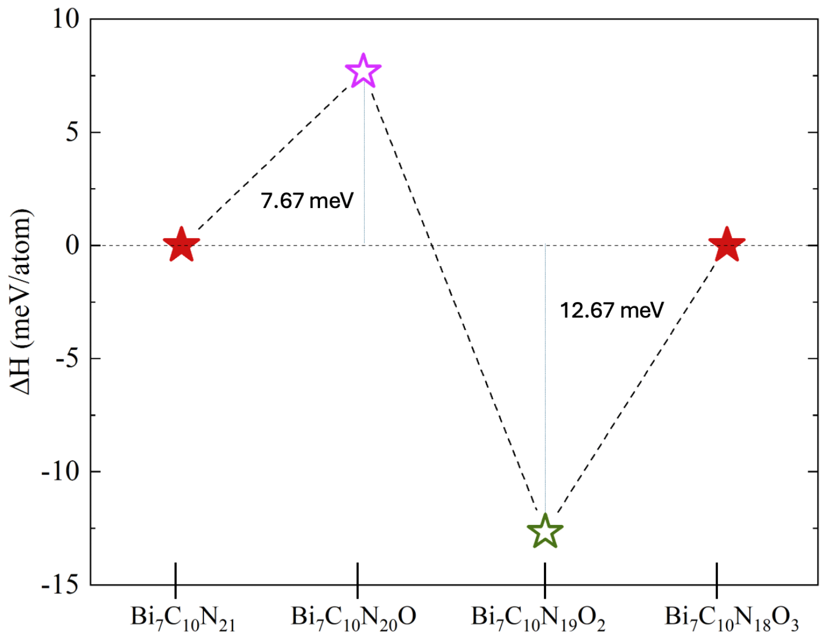

$$\Delta H_{mix} = [\, E(Bi_7C_{10}N_{18}(N_{3(1-x)}O_{3x}) - A{*}E(Bi_7C_{10}N_{21}) - B{*}E(Bi_7C_{10}N_{18}O_3)\,]$$

A=1 , B=0 for $Bi_7C_{10}N_{21}$ (H_{mix}=0)

A=2/3 , B=1/3 for $Bi_7C_{10}N_{20}O_1$

A=1/3 , B=2/3 for $Bi_7C_{10}N_{19}O_2$

A=0 , B=1 for $Bi_7C_{10}N_{18}O_3$ (H_{mix}=0)

Figure S4: Relative enthalpy difference with respect to the end members, $Bi_7C_{10}N_{21}$ & $Bi_7C_{10}N_{18}O_3$. An energy difference of < 25 meV suggests that all four structural models are possible at room temperature.

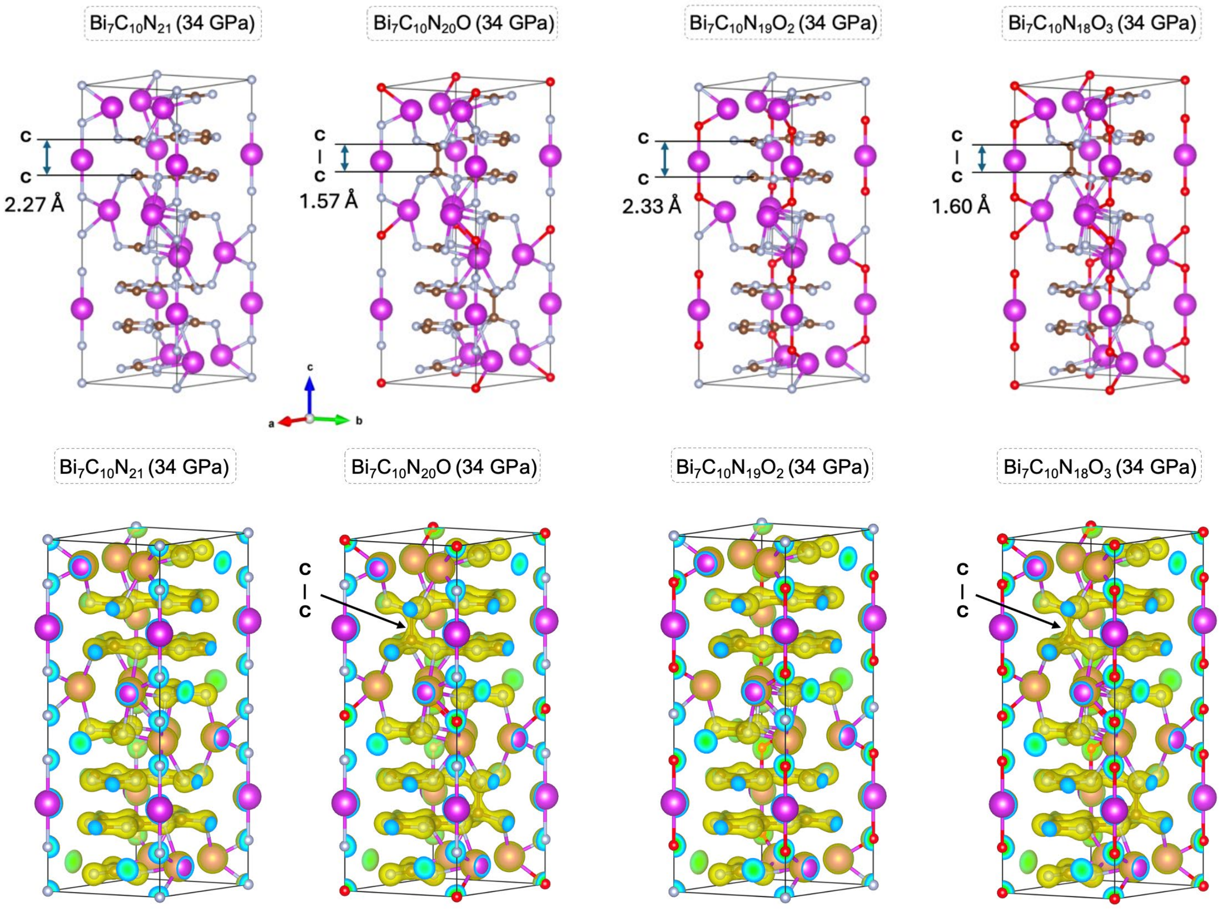

Figure S5: DFT optimized crystal structure of pure $Bi_7C_{10}N_{21}$, and oxygen substituted phases ($Bi_7C_{10}N_{20}O$, $Bi_7C_{10}N_{19}O_2$, and $Bi_7C_{10}N_{18}O_3$) at a pressure of 34 GPa. The corresponding charge density isosurfaces with isosurface level 0.2 e/Å^3.

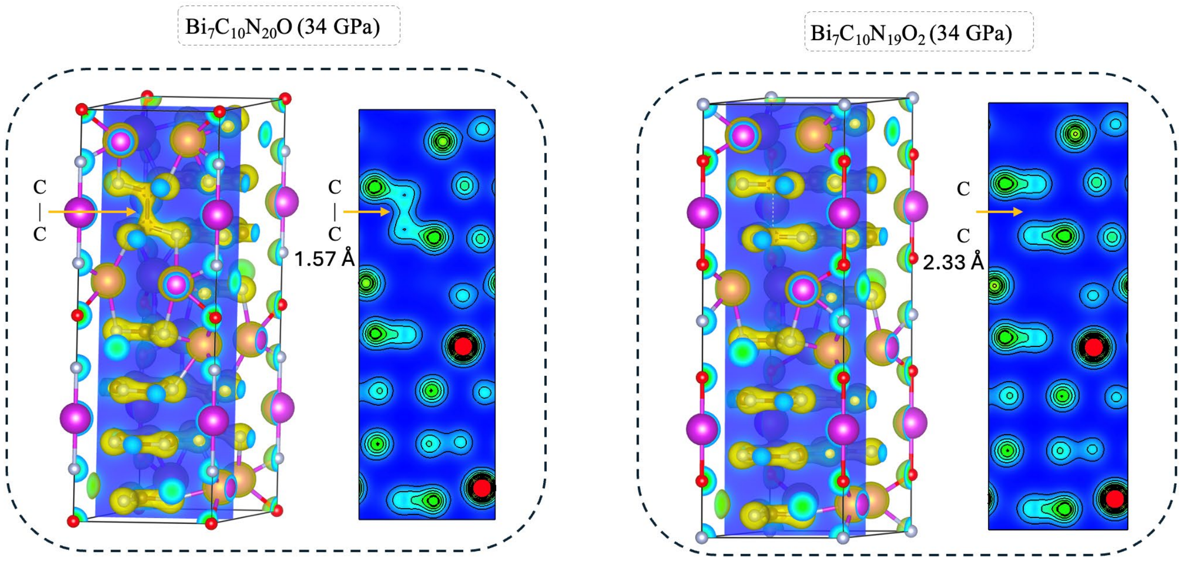

Figure S6: 2D planar projection of the charge density isosurface along the plane passing through one of the C-C lateral bond for $Bi_7C_{10}N_{20}O$, and $Bi_7C_{10}N_{19}O_2$ at 34 GPa, respectively.

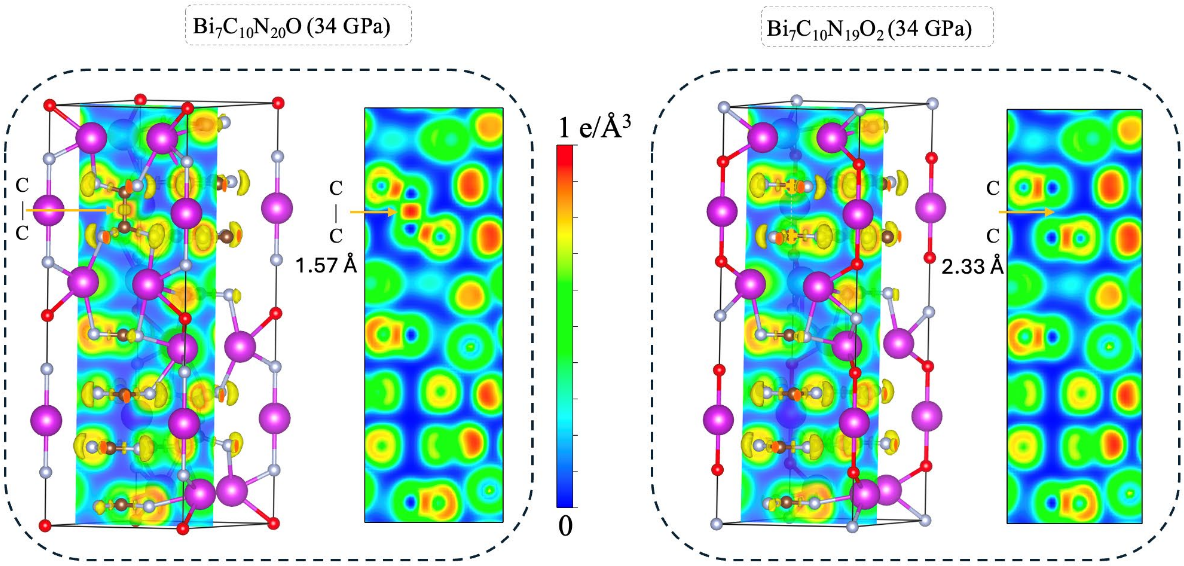

Figure S7: Electron localization function (ELF) and a planar projection of ELF isosurface along the plane passing through one of the C-C lateral bond for $Bi_7C_{10}N_{20}O$, and $Bi_7C_{10}N_{19}O_2$ at 34 GPa, respectively.

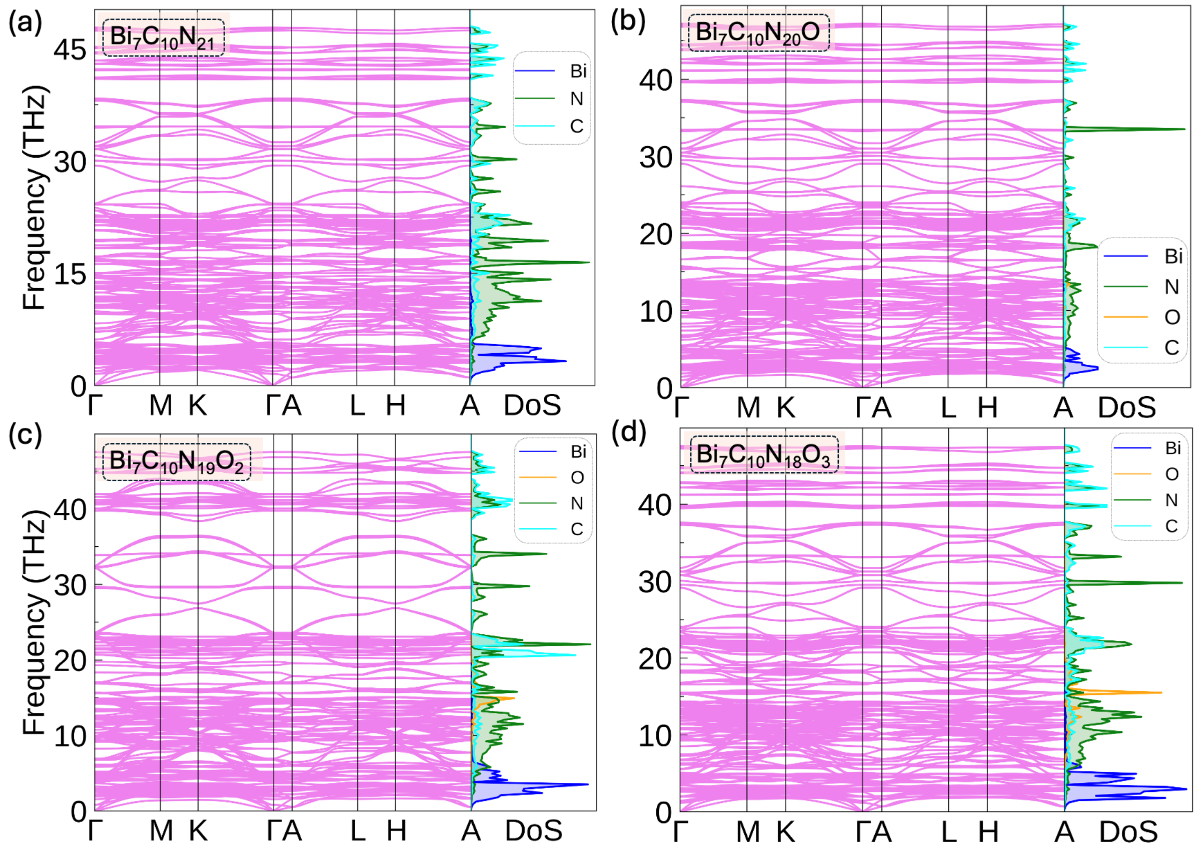

Figure S8: Phonon dispersion relations calculated for all the considered structural models at a pressure of 34 GPa.

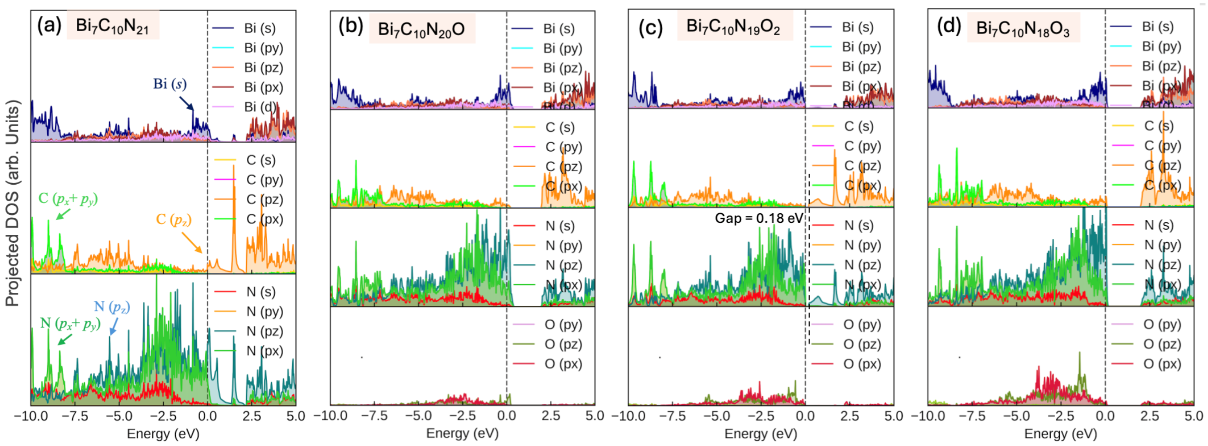

Figure S9: Electronic density of states (EDOS) projected onto different chemical elements and corresponding atomic orbitals for all the considered structural models calculated at a pressure of 34 GPa.

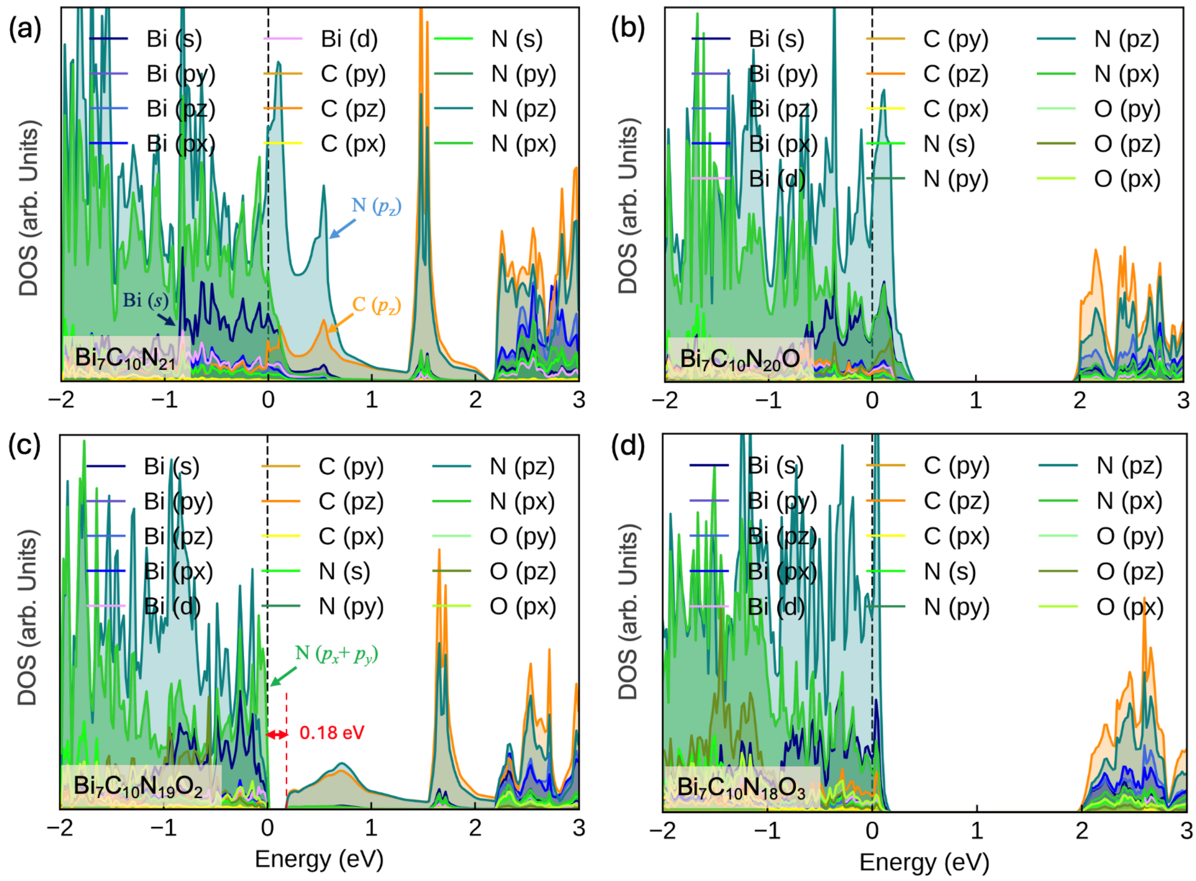

Figure S10: Electronic density of states (EDOS) projected onto different chemical elements and corresponding atomic orbitals for all the considered structural models in an energy window of -2 to 3 eV around the Fermi energy calculated at a pressure of 34 GPa.

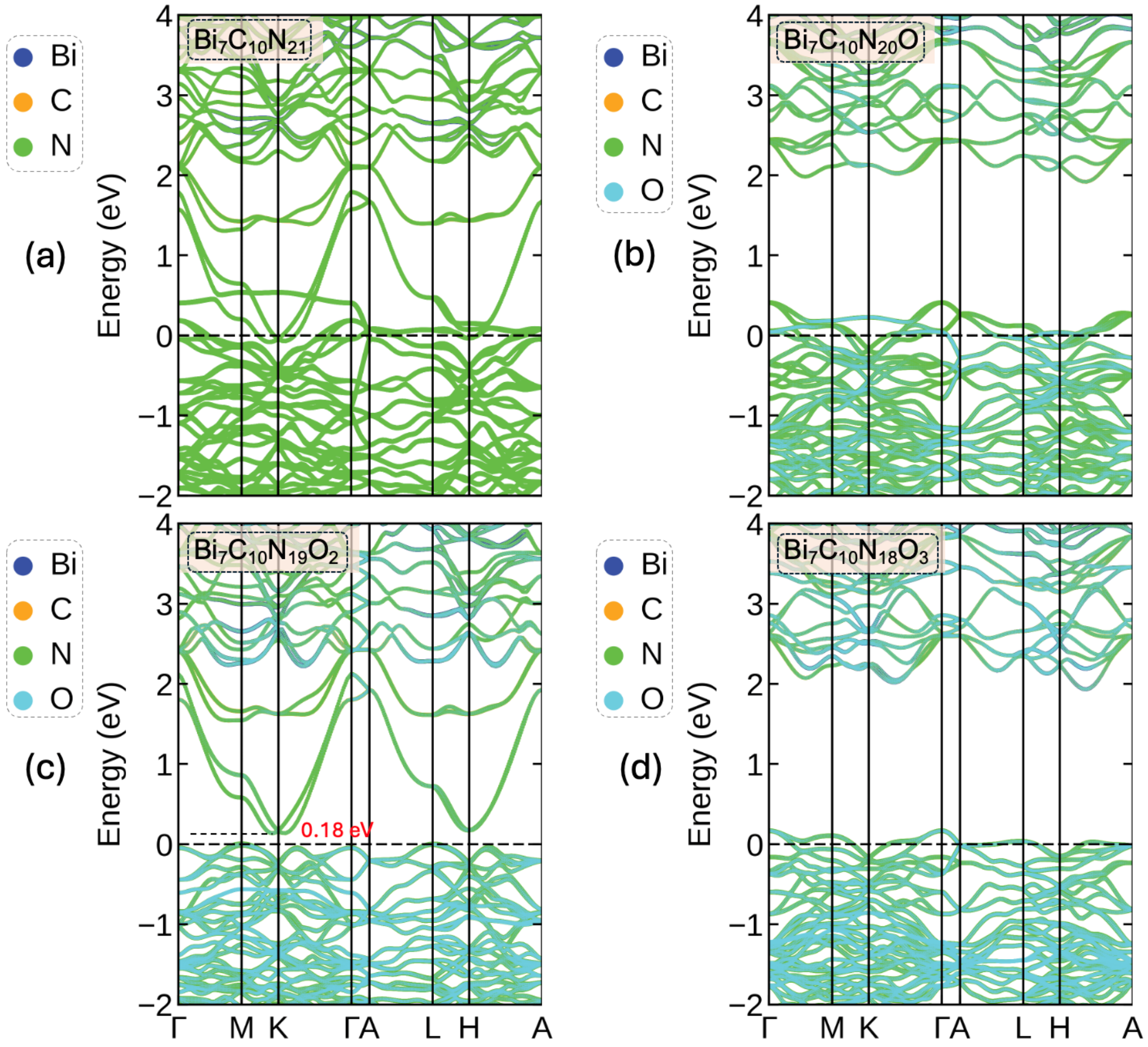

Figure S11: Electronic band structure of $Bi_7C_{10}N_{21}$, $Bi_7C_{10}N_{20}O$, $Bi_7C_{10}N_{19}O_2$, and $Bi_7C_{10}N_{18}O_3$ calculated at a pressure of 34 GPa.

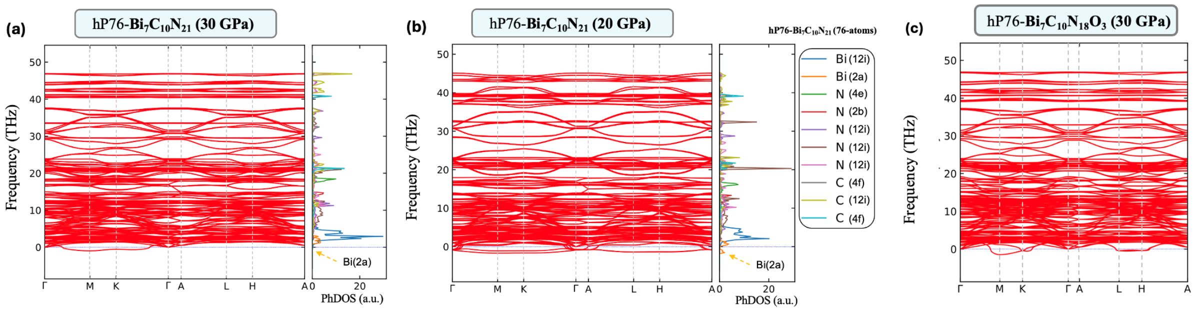

Figure S12: (a)-(b) Phonon dispersion relations calculated for $Bi_7C_{10}N_{21}$ at 30 GPa, and 20 GPa, respectively. (c) Phonon dispersion relations calculated for $Bi_7C_{10}N_{18}O_3$ at 30 GPa.
